# Stromal Integrin α11β1 Affects RM11 Prostate and 4T1 Breast Xenograft Tumors Differently

**DOI:** 10.1371/journal.pone.0151663

**Published:** 2016-03-18

**Authors:** Inga Reigstad, Hilde Y. H. Smeland, Trude Skogstrand, Kristina Sortland, Marei Caroline Schmid, Rolf K. Reed, Linda Stuhr

**Affiliations:** 1 Department of Biomedicine, University of Bergen, Bergen, Norway; 2 Matrix biology group, Haukeland University Hospital, Bergen, Norway; 3 Center of Cancer Biomarkers, University of Bergen, Bergen, Norway; University of Patras, GREECE

## Abstract

**Purpose:**

It has been implied that the collagen binding integrin α11β1 plays a role in carcinogenesis. As still relatively little is known about how the stromal integrin α11β1 affects different aspects of tumor development, we wanted to examine the direct effects on primary tumor growth, fibrosis, tumor interstitial fluid pressure (PIF) and metastasis in murine 4T1 mammary and RM11 prostate tumors, using an *in vivo* SCID integrin α11-deficient mouse model.

**Methods:**

Tumor growth was measured using a caliper, PIF by the wick-in-needle technique, activated fibroblasts by α-SMA immunofluorescence staining and fibrosis by transmission electron microscopy and picrosirius-red staining. Metastases were evaluated using hematoxylin and eosin stained sections.

**Results:**

RM11 tumor growth was significantly reduced in the SCID integrin α11-deficient (α11-KO) compared to in SCID integrin α11 wild type (WT) mice, whereas there was no similar effect in the 4T1 tumor model. The 4T1 model demonstrated an alteration in collagen fibril diameter in the integrin α11-KO mice compared to WT, which was not found in the RM11 model. There were no significant differences in the amount of activated fibroblasts, total collagen content, collagen organization or PIF in the tumors in integrin α11-deficient mice compared to WT mice. There was also no difference in lung metastases between the two groups.

**Conclusion:**

Deficiency of stromal integrin α11β1 showed different effects on tumor growth and collagen fibril diameter depending on tumor type, but no effect on tumor PIF or development of lung metastasis.

## Introduction

Carcinomas consist of both malignant cells and stroma, the latter being composed of extracellular matrix (ECM) molecules and associated cells [1]. The main focus of cancer research has traditionally been on tumor cell alterations, but in the last decade ECM has been identified as an important contributor to tumor development and progression [2, 3]. Tumor cells release growth factors and proteolytic enzymes that modulate the stroma [4, 5], and the stromal components interact with the tumor cells in a reciprocal manner to regulate different aspects of tumor development [1].

Integrins belong to a family of major cell surface receptors that mainly bind ECM proteins. Twenty-four different integrins, forming heterodimers by combining 18 α- and 8 β-subunits, have been identified. The integrins mediate cell-cell and cell-matrix adhesion and are capable of cell inside-out and outside-in signaling [6, 7]. Their role on the surface of tumor cells has been extensively studied, and integrins contribute to proliferation, migration and survival of malignant cells, and have thus been suggested to play an important role in tumor progression [8]. Altered integrin expression in several stromal cells, including the cancer associated fibroblasts, may also influence tumor growth and progression [8–10], and the present study focuses on the effect of the collagen binding integrin α11β1 in the tumor stroma.

The integrin subunit α11 forms a heterodimer with integrin subunit β1 and is one of four collagen-binding integrins [11]. In mouse embryos, integrin α11β1 is a major collagen receptor on a subset of fibroblasts [12], but characterization of its expression in adult and human tissue is still insufficient [13]. Integrin α11β1 has high affinity for collagen type I, and has been indicated to be involved in cell migration and collagen reorganization [10, 14, 15], but other than this, there is limited knowledge about α11β1’s normal physiological role.

Integrin α11 has also been implicated to play a role in carcinogenesis. Integrin α11 is expressed in metastases from human malignant melanoma [16], and in stromal fibroblasts in human non-small-cell lung cancer (NSCLC) [9, 17]. It has also been shown, both *in vitro* and *in vivo*, that α11 integrin expressed on fibroblasts may stimulate the growth of tumor cells [9, 10, 18]. Earlier findings have indicated that stromal integrin α11 has a role in both primary tumor growth and in the metastatic process [9, 10], and this has raised the question if integrin α11 could be used as a biomarker, or if targeting integrin α11 could prove to be a novel approach in cancer treatment.

Since still relatively little is known about how the stromal integrin α11β1 in tumors affects different aspects of tumor development, we decided to examine the direct effects of integrin α11β1 on primary tumor growth, fibrosis, tumor interstitial fluid pressure and metastasis in a 4T1 mammary tumor- and a RM11 prostate tumor model.

## Methods

### Cell Lines

The murine mammary carcinoma cell line 4T1 was obtained from the American Type Culture Collection (Manassas, VA., USA). The prostate cell line RM11 was a gift from Associate professor Thomas S. Griffith (University of Minnesota, Minneapolis, MN., USA). This cell line was originally derived from a ras/myc reconstituted tumor in a Balb/c mouse [19]. The cells were grown in RPMI-1640 medium (HEPES solution for RM11 cells) supplemented with 10% Foetal Bovine Serum (Sigma-Aldrich, Steinheim, Germany), 100 units/ml penicillin, 100 μg/ml streptomycin, 1–2% L-glutamine (all from Sigma-Aldrich, Steinheim, Germany), with an addition of 1% sodium pyruvate for the RM11 cells. All cells were grown as single monolayers in a humidified incubator at 37°C in 5% CO_2_ and they were seeded and used at log phase in all experiments. SV40 transformed wild type MEF cell line [12] was cultured in DMEM (Sigma-Aldrich, Steinheim, Germany), 10% Foetal Bovine Serum and 100 units/ml penicillin, 100 μg/ml streptomycin as previosly described.

### Animal Model

The integrin α11-deficient heterozygous SCID mouse strain was generated as described [10]. The mice were bred heterozygously, and SCID integrin α11 wild type (WT) and SCID integrin α11-deficient (α11-KO) offsprings were used in the experiments. PCR-genotyping was performed on DNA extracted from ear biopsies as previously described [20]. Female mice were used for the mammary 4T1 tumor model and male mice for the prostate RM11 tumor model. The animals were kept in individually ventilated cages and cared for regularly. Efforts were made to age- and weight match the animals. The animal experiments were approved by the local ethical committee at The Laboratory Animal Facility, the Department of Clinical Medicine, the Faculty of Medicine and Dentistry, University of Bergen (Permit Number 20135571). All experiments were performed in accordance with the regulations of the Norwegian Animal Research Authority.

### Establishing Tumors

A total of 1 x 10^6^ 4T1 tumor cells in 0.15 ml PBS were injected into the fourth mammary fat pads on each side. 3 x 10^5^ RM11 cells were injected subcutaneously on both sides of the mouse flank. The 4T1 tumors were measured using a caliper on days 7, 10, 13, 16 and endpoint 18 post injection, but some were ended day 17 due to rapid tumor growth. The RM11 tumors were measured using a caliper on days 9, 11 and endpoint day 13 post injection. All experiments were performed blinded to genotype. The tumor volume was calculated using the formula; *tumor volume (mm*^*3*^*)* = *(π/6)* x *a*^*2*^ x *b*, where *a* represents the shortest diameter and *b* represents the longest diameter of the tumor. All animals were anesthetized using Isofluran (Isoba®vet. 100%, Schering-Plough A/S, Farum, Denmark) and were sacrificed by cervical dislocation under anesthesia.

### Measurement of Interstitial Fluid Pressure

The tumor interstitial fluid pressure (PIF) was measured using the wick-in-needle technique [21]. Briefly, a standard 23-gauge needle with a side hole filled with nylon floss and saline was inserted into the central part of the tumor after calibration and connected to a PE-50 catheter, a pressure transducer and a computer for pressure registrations, using the software Powerlab chart (version 5, PowerLAb/ssp ADinstruments, Dunedin, New Zealand). After a period of stable pressure measurements, the fluid communication was tested by clamping the catheter which shall cause a transient rise and fall in pressure. Measurements were accepted if the pre- to post-clamping value was within ± 1 mmHg.

### Electron Microscopy of Collagen Fibrils in the Tumor

A JEM-1230 Transmission Electron Microscope (TEM) (Jeol, Tokyo, Japan) was used to measure the diameter of the collagen fibrils. The tissue samples were cut into approximately 1x1x1 mm samples and fixed in 2.5% glutaraldehyde in 0.1 M phosphate buffer, and then washed in PBS. The samples were post-fixed in 1% OsO_4_ in PBS and dehydrated in increasing concentrations of ethanol (70%, 95% and 100%), and then propylenoxide, before being embedded in Agar 100 Resin and sectioned at 60 nm. Four to five images from different areas of the tissue were captured at x100 000 magnification and analyzed using Image J 1.46 (National Institute of Health, Beteshda, MD., USA). Because of uneven distribution of collagen in the tissue, the images were taken from the areas of the tissue where collagen was found.

A Jeol JSM-7400F Scanning Electron microscope (SEM) was used to study the tumor collagen scaffold architecture. The tumors were cut in 1x1x1 mm samples and fixed in 2.5% glutaraldehyde in 0.1 M phosphate buffer, before being placed in 10% NaOH for 7 days with replacement every day. The samples were thereafter placed in tap-water for 2–4 days and then dehydrated in increasing concentrations of ethanol (70%, 95% and 100%), and dried in a “critical point-dryer”. The tumor tissue was mounted on an Au-stub and coated with a 10 nm layer of gold and palladium using a Jeol JFC-2300HR High Resolution fine coater. Five images from different areas of the tumor were captured from each tumor at x10 000 magnification.

### Protein Extraction and Western Blot Analysis

The protein expression of integrin α11 in tumors lysates and cultured tumor cell lines was investigated. Tumor samples and cultured cell lines were lysed in buffer containing 50 mM Tris-HCl, pH 7.4, 150 mM NaCl and 1% Triton X supplemented with one tablet of protease inhibitor cocktail (complete EDTA-free; Roche Diagnostics GMBh, Mannheim, Germany) per 10 ml buffer. After homogenization, tumor samples were centrifuged for 10 min at 12 000 rpm and protein concentration was measured using Pierce^TM^ BCA Protein Assay Kit (Life Technologies, Thermo Fisher Scientific, Waltham, MA., USA) according to the manufacturer’s protocol. For western blot analysis cell lines were grown to confluency in 6 well plates washed with cold PBS, lysed and scraped with cell scraper on ice. Cell lysates were centifuged at 13 000 rpm for 30 min at +4° C, and supernatant harvested. Protein lysates were loaded in XT Sample Buffer, 4X (Bio-Rad Laboratories, Inc, Hercules, CA., USA) containing 50 mM DL-Dithiothreitol (dTT) (Sigma-Aldrich, Steinheim, Germany), and run through a 10% Precise^TM^ Protein Gel (Life Technologies, Thermo Fisher Scientific). The proteins were then transferred to a nitrocellulose membrane using Invitrogen^TM^ iBlot® Dry Blotting System (Life Technologies, Thermo Fisher Scientific) and an iBlot® Transfer Stack (Life Technologies, Thermo Fisher Scientific). After blocking in I-block (Life Technologies, Thermo Fisher Scientific) for 1 hour at room temperature, the membranes were incubated over-night with rabbit polyclonal anti-mouse α11 antiserum [22] 1:500 in I-block at +4°C. The anti-mouse α11 antiserum is produced against the peptide CRREPGLDPTPKVLE from the integrin α11 cytoplasmic domain (Innovagen AB, Lund, Sweden) [22]. This was followed by incubation with a HRP-coupled secondary antibody (goat anti-rabbit; AB97051, Abcam, Cambridge, UK; 1:5000 in TBS-T). The bands were visualized by the ECL system Pierce^TM^ ECL Western Blotting Substrate (Life Technologies, Thermo Fisher Scientific). The membrane was then re-probed with β-actin antibody (AB8227, Abcam; 1:5000 in I-block) and HRP-coupled secondary antibody (goat anti-rabbit; AB97051, Abcam; 1:5000 in TBS-T). Membranes were visualized using the Gel ChemiDoc system and Quantity One 4.6.6 imaging software (Bio-Rad Laboratories, Inc, Hercules, CA., USA).

### Picrosirius-Red and Immunofluorescence Staining

For a semi-quantitative measurement of collagen type I and III, picrosirius-red stain (Polysciences inc, Warrington, FL., USA) was used. Five paraffin embedded tumor sections with a thickness of 5μ from each group were deparaffinized, stained in picrosirius-red for one hour, dehydrated and mounted. Five to six images from each tumor were captured with x10 magnification (Nikon Digital Sight, Nikon Corporation, Tokyo, Japan).

For α-SMA staining, FITC-conjugated monoclonal anti-actin α-smooth muscle antibody (F3777, dilution 1:200, Sigma Aldrich, Steinheim, Germany) was used. Five paraffin embedded tumor sections with a thickness of 10μ from each group were stained. Prior to staining, the sections were first deparaffinized, and then placed in citrate buffer for 25 minutes in 95°C. Non-specific background staining was reduced by adding 0.3% hydrogen peroxide in methanol to the sections. Five images from each tumor were captured with x20 magnification with an Axioscope fluorescence microscope and a digital Axiocam MRm camera (Zeiss, Oberkochen, Germany).

To identify the amount of pixels positive for picrosirius-red staining and α-SMA, the software Image J (National Institute of Health, Beteshda, MD., USA) was used. Individual threshold values were set for each image to adjust for differences in intensity and background. For both stainings, images were taken in an organized pattern in the tumor periphery in order to avoid the necrotic central area.

### Metastasis

To allow development of metastasis, female animals were injected with 3 x 10^5^ 4T1 cells in their right, fifth mammary fat pad. The experiment was ended on day 21 due to animal welfare. The lungs were fixed using approximately 1 ml of Bouin’s solution (Gurr BDH Chemicals Ltd, Poole, UK) injected into the trachea. Then the lungs were dissected out, fixed in new Bouin’s solution and washed in 70% ethanol before dehydration. Immediately following this procedure, the liver and brain were harvested and fixed in formalin. All tissues were embedded in paraffin using standard procedures, sectioned, and stained with hematoxylin and eosin (H & E). To quantify lung metastasis, three coronal sections, 600 μm apart and covering both lungs, were examined for each animal. The number of metastases per section was counted and total area covered by metastases was measured (Nikon Digital Sight, Nikon Corporation, Tokyo, Japan).

### Statistical Analysis

For statistical analysis, Sigmaplot 12.5 (Systat Software Inc., Chicago, IL., USA) and Graph Pad Prism 6 (GraphPad Software, Inc., La Jolla, CA., USA) were used. Either the unpaired two-tailed t-test or the Mann U Whitney t-test, were used to analyze statistical differences between the two groups. For analysis of tumor growth, t-test with Welch correction was used. The mice injected with 4T1 cells were sacrificed at either day 17 or day 18, and the tumors harvested on the same day were tested against each other. Results were accepted as statistically different when p < 0.05. Graph Pad Prism 6 was used to create all figures. Data is given as mean ± SD, and number of measurements (n) refers to number of tumors unless otherwise specified.

## Results

### Integrin α11 Expression

**Fig 1:**
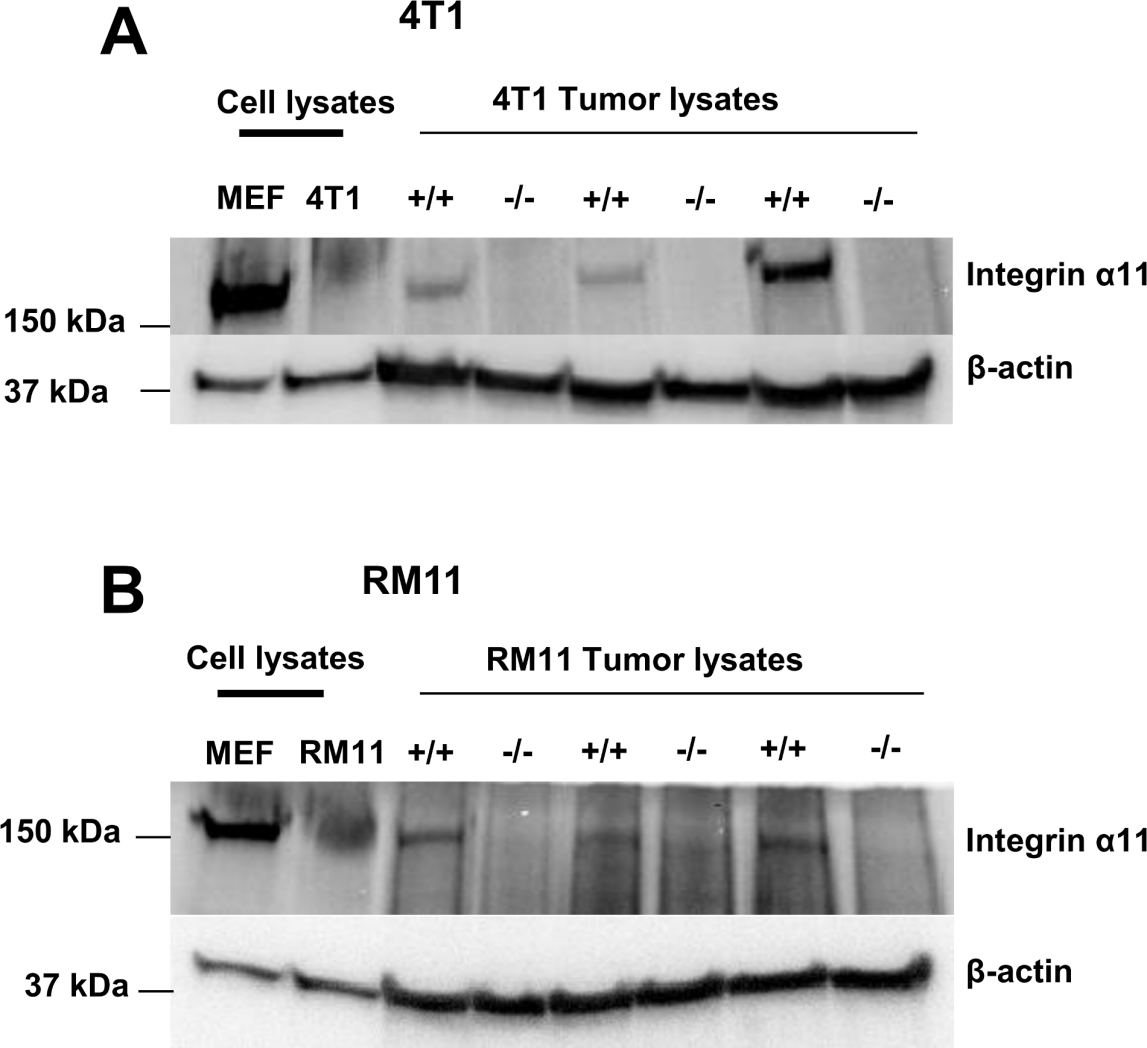
The integrin α11 expression in 4T1 (A) and RM11 (B) cultured tumor cells and tumor lysates from tumors in SCID integrin α11 wild type (+/+) and SCID integrin α11-deficient (-/-) mice. Positive control is a SV40 transformed wildtype mouse embryonic fibroblast cell line (MEF).

Using western blotting, integrin α11 was found to be expressed in tumor lysates from both 4T1 and RM11 tumors grown in WT mice, whereas no integrin α11 was detected in the tumors grown in α11-KO mice (Fig 1). No integrin α11 expression was detected in lysates from cultured 4T1 and RM11 tumor cells (Fig 1).

### Tumor Growth

**Fig 2:**
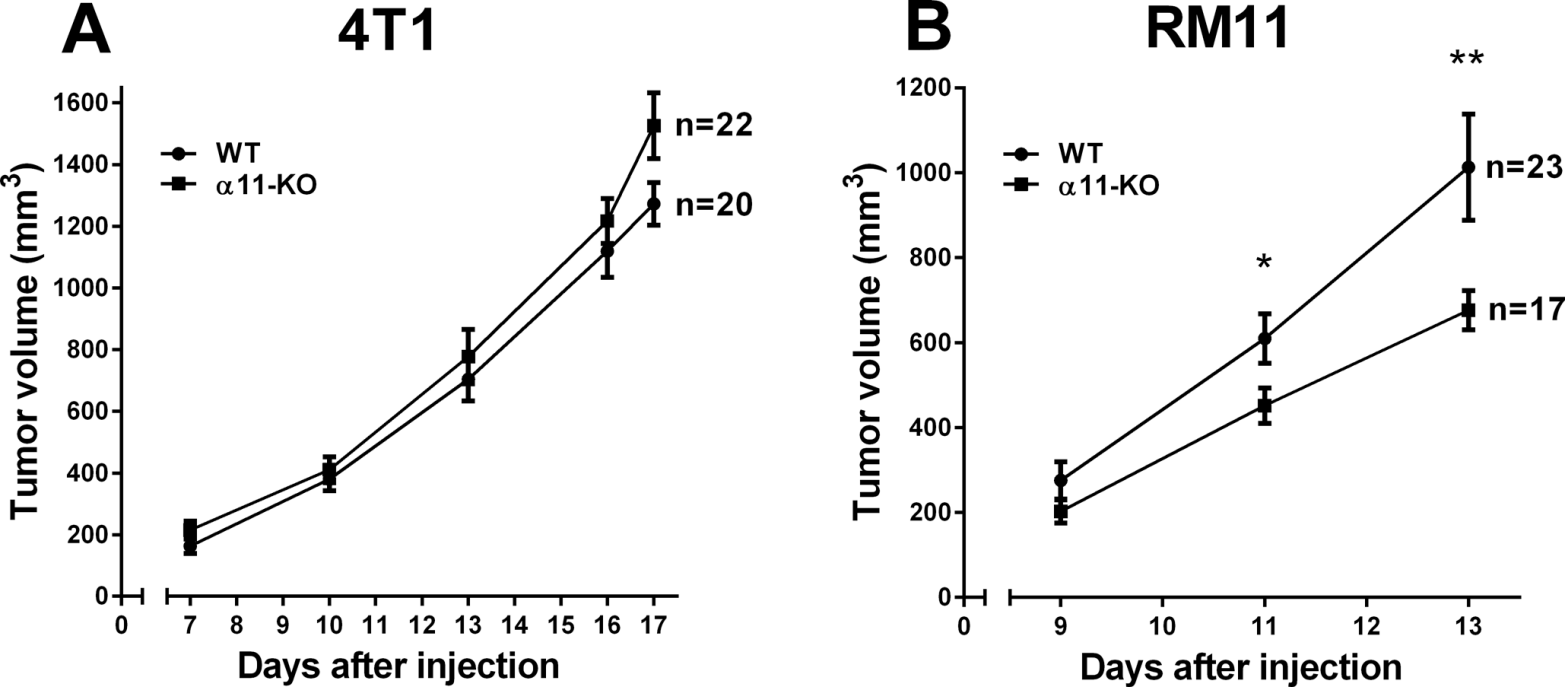
The growth of 4T1 (A) and RM11 (B) tumors in SCID integrin α11 wild type (WT) and SCID integrin α11-deficient (α11-KO) mice. A total of 1 x 10^6^ 4T1 and 3 x 10^5^ RM11 cells were injected into the mammary fat pad and subcutaneously in the mouse flank, respectively. Endpoint values in the 4T1 model include tumors that were harvested on days 17 and 18. Mean ± SEM. * p < 0.04, ** p < 0.02.

The tumor volume of RM11 prostate tumors grown subcutaneously were significantly impeded (p < 0.04 and 0.02) in tumors grown in α11-KO mice compared to tumors grown in WT mice during their 13 day growth period, whereas 4T1 mammary tumors did not show any difference in tumor growth between α11-KO mice and WT mice when comparing tumors that were harvested on the same day (17 or 18) (Fig 2). All end-point measurements in the 4T1 tumor model are summarized at day 17 in Fig 2.

### α-Smooth Muscle Actin (α-SMA) Expression

**Fig 3:**
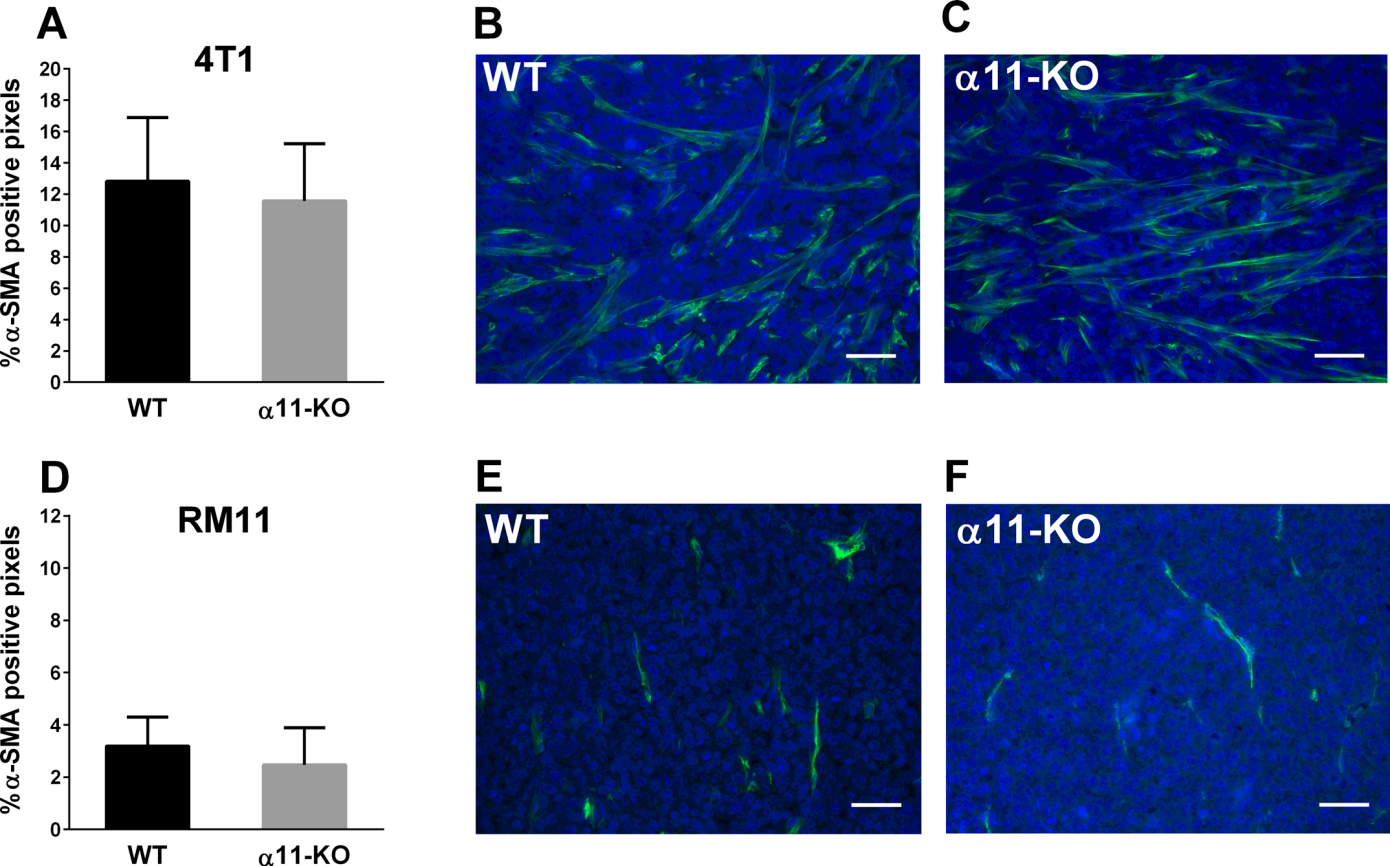
Percentage of pixels positive for α-SMA in 4T1 (n = 5) and RM11 (n = 5) tumors from SCID integrin α11 wild type (WT) and SCID integrin α11-deficient (α11-KO) mice were calculated from immunofluorescent images (A, D). No statistical differences in 4T1 (p = 0.62) or RM11 (p = 0.40) tumors were found using unpaired two-tailed t-test. Mean ± SD. Representative images of α-SMA-staining (green) from both genotypes in 4T1 (B, C) and RM11 (E, F) tumors are shown. Scale bars indicate 50 μm.

α-SMA immunofluorescent stained tumor sections were used to quantify the relative amount of activated fibroblasts in the tumors (represented by pixels). There was no significant difference in the level of α-SMA expression in the 4T1 or the RM11 tumors grown in α11-KO mice compared to that in WT mice (Fig 3).

### Tumor Collagen Structure

**Fig 4:**
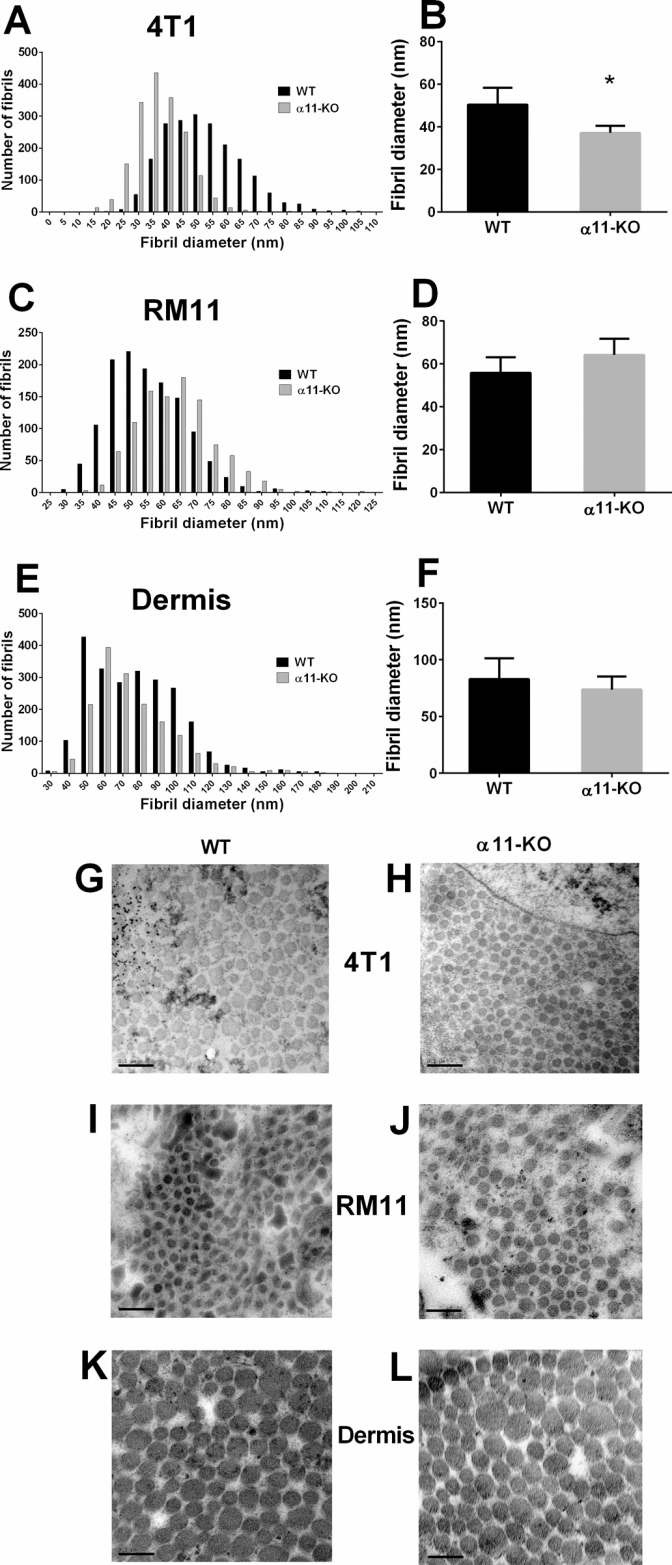
Collagen fibrils were analyzed using transmission electron microscopy (TEM). Collagen fibril diameter distribution and average fibril diameter per tumor in 4T1 (n = 7 and n = 5) tumors (A, B), showed a shift towards thinner fibrils in SCID integrin α11-deficient (α11-KO) mice. RM11 tumors (n = 4 and n = 3) (C, D) and dermis (n = 4 and n = 3) (E, F) showed no significant differences in average collagen fibril diameter in SCID integrin α11 wild type (WT) and SCID integrin α11-deficient (α11-KO) mice (RM11 p = 0.20, dermis p = 0.47) using unpaired two-tailed t-test. Mean ± SD. * p < 0.006. Representative TEM images of collagen fibrils from both genotypes in 4T1 tumors (G, H), RM11 tumors (I, J) and dermis (K, L) are shown. Scale bars indicate 0.2 μm.

In the present study collagen structure in the α11-KO versus WT tumors was compared by measuring the collagen fibril diameters using transmission electron microsopy (TEM) analyses. An uneven distribution of fibril diameter was found, leading to a shift towards thinner collagen fibrils in 4T1 carcinomas grown in α11-KO mice compared to WT (Fig 4A). The average collagen diameter in the 4T1 tumors grown in α11-KO mice (37.2 ± 1.5 nm) was significantly smaller (p < 0.006) than in WT mice (50.4 ± 3.0 nm) (Fig 4B). In the RM11 tumors there was no such difference in the collagen diameter between the tumors grown in α11-KO and WT mice (Fig 4D). To evaluate whether the decreased collagen fibril diameter was a more general feature in the α11-KO mice and not only tumor specific, collagen fibril diameter in dermis from healthy male mice was evaluated. In dermis, no difference in the collagen diameter between α11-KO and WT mice was found.

**Fig 5:**
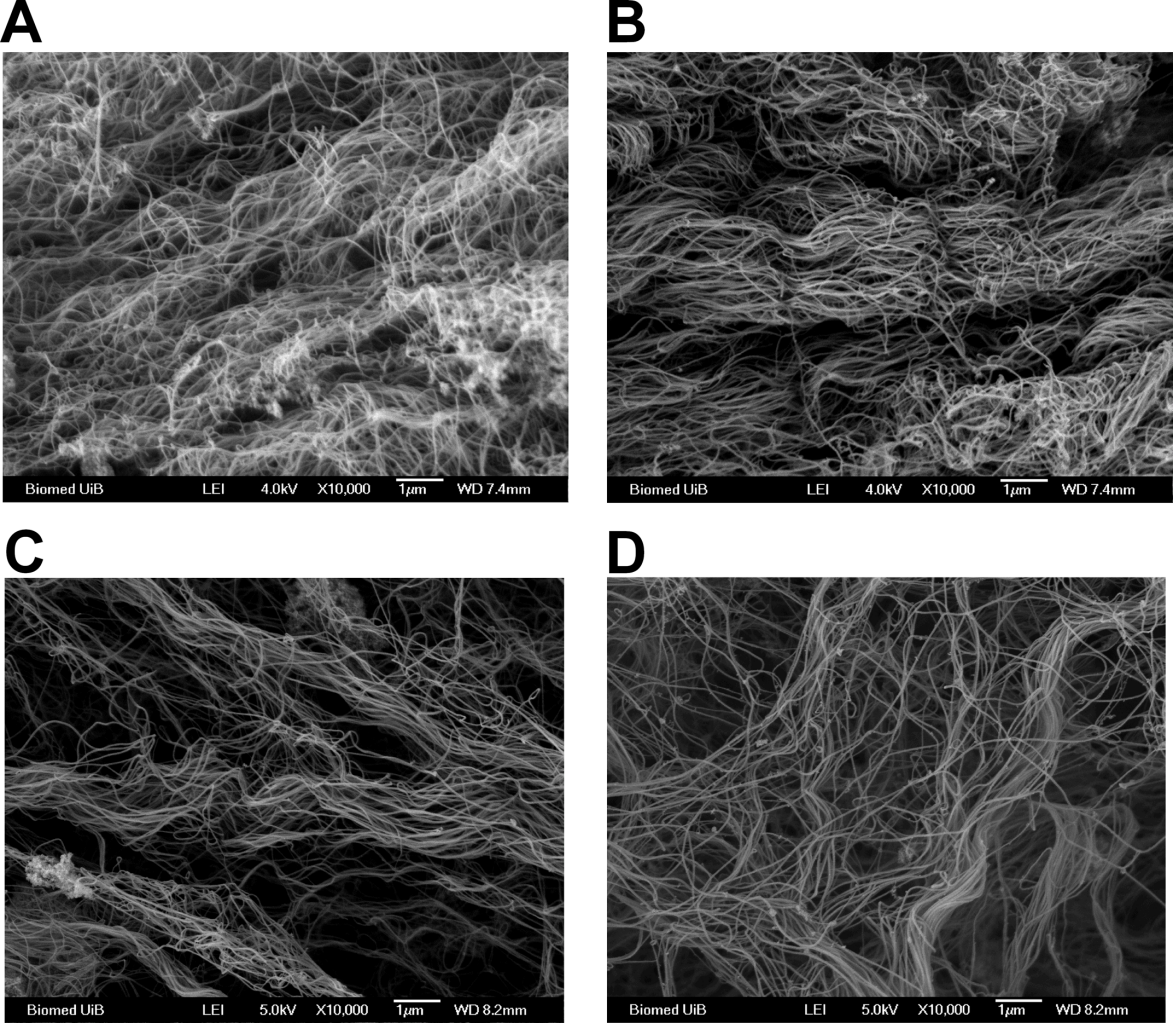
A representative scanning electron micrograph of collagen in 4T1 tumors (n = 3) from SCID integrin α11 wild type (WT) (A) and SCID integrin α11-deficient (α11-KO) (B) and RM11 tumors (n = 3) from WT (C) and α11-KO mice (D), respectively. Scale bars indicate 1 μm.

Scanning electron microscopy (SEM) did not show any difference in collagen architecture between the α11-KO and WT neither in the 4T1 tumors nor the RM11 tumors (Fig 5).

### Tumor Collagen Amount

**Fig 6:**
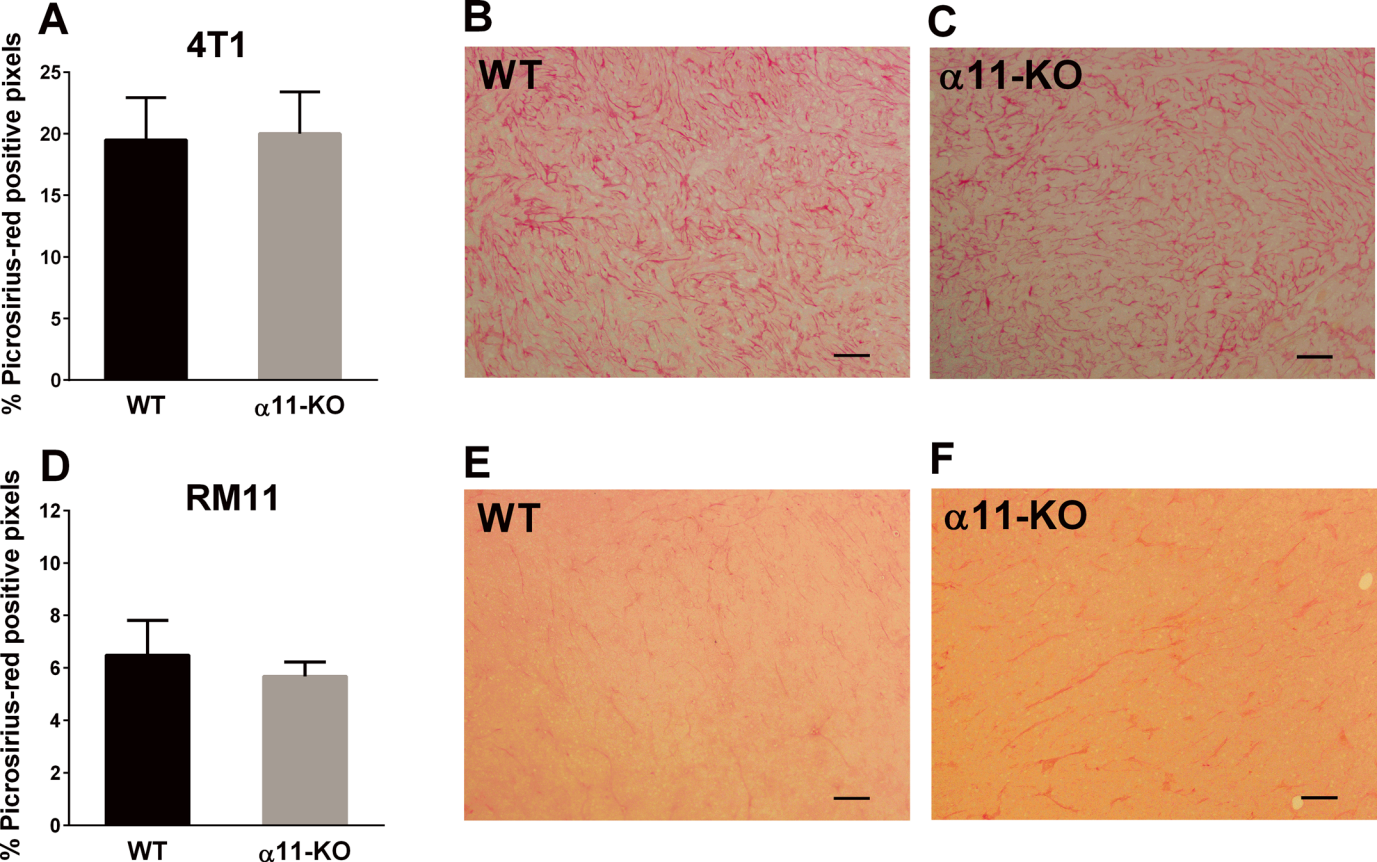
The total fraction of picrosirius-red staining quantified in 4T1 (n = 5) and RM11 (n = 5 and n = 3) tumors in SCID integrin α11 wild type (WT) and SCID integrin α11-deficient (α11-KO) (A, D). No statistical differences in 4T1 (p = 0.82) or RM11 (p = 0.37) tumors were found using unpaired two-tailed t-test. Mean ± SD. Representative images of picrosirius-red staining from both genotypes in 4T1 (B, C) and RM11 (E, F) tumors are shown. Scale bars indicate 100 μm.

Picrosirius-red staining was used to quantify the most abundant collagens; type I and III, in the tumor sections. No significant difference in the amount of collagen was seen in the 4T1 and the RM11 tumors grown in α11-KO compared to those grown in WT mice (Fig 6).

### Tumor Interstitial Fluid Pressure (PIF)

**Fig 7:**
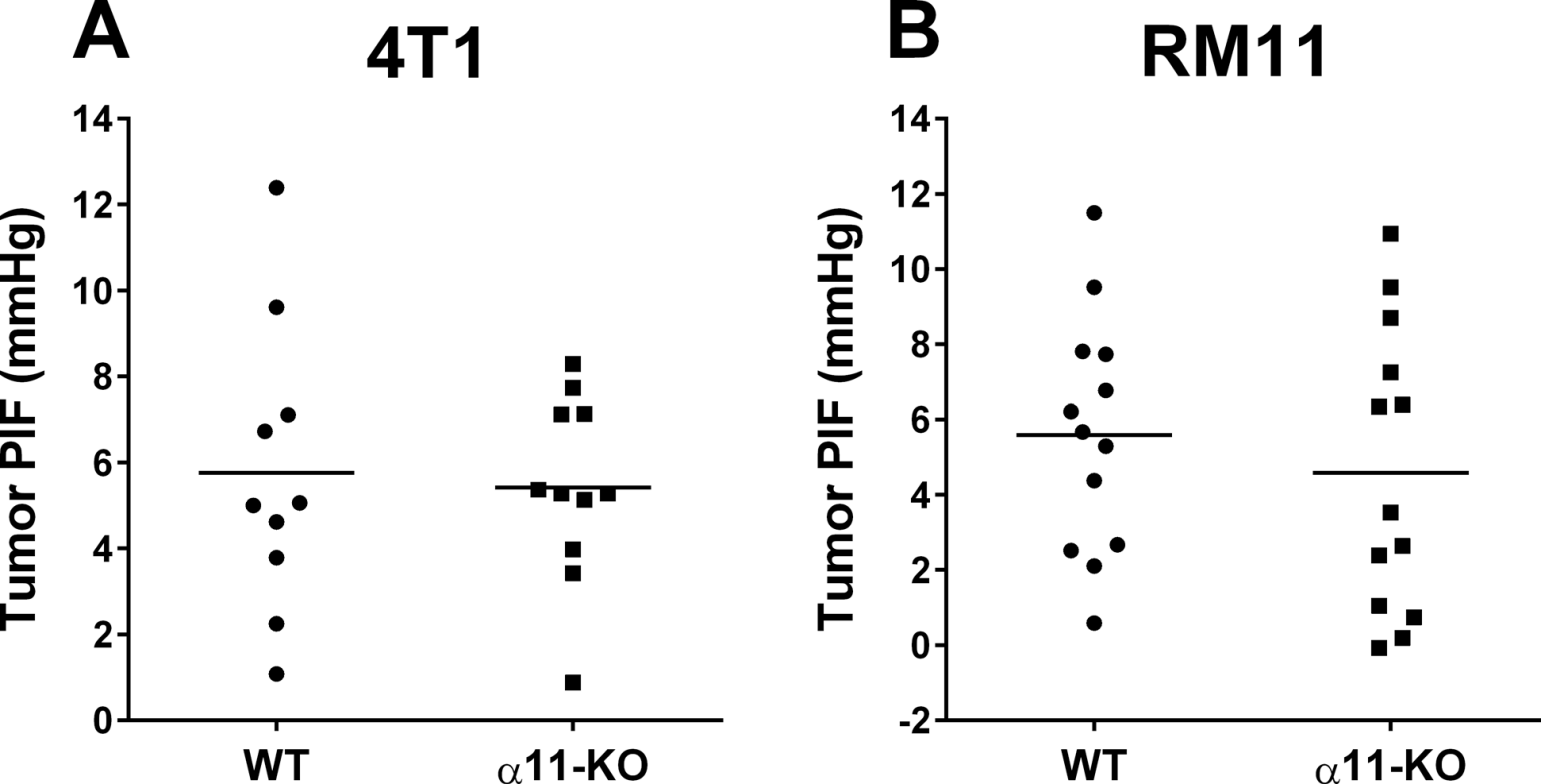
The individual interstitial fluid pressures (PIF) in 4T1 (A) and RM11 (B) tumors in SCID integrin α11 wild type (WT) and SCID integrin α11-deficient (α11-KO) mice. The horizontal lines indicate the mean values. No statistical differences in 4T1 (p = 0.78) or RM11 (p = 0.47) tumors were found using unpaired two-tailed t-test.

Tumor PIF was determined using the wick-in-needle technique. There was no significant difference in PIF in either 4T1 or RM11 tumors grown in α11-KO versus WT mice (Fig 7).

### Tumor Metastases

**Fig 8:**
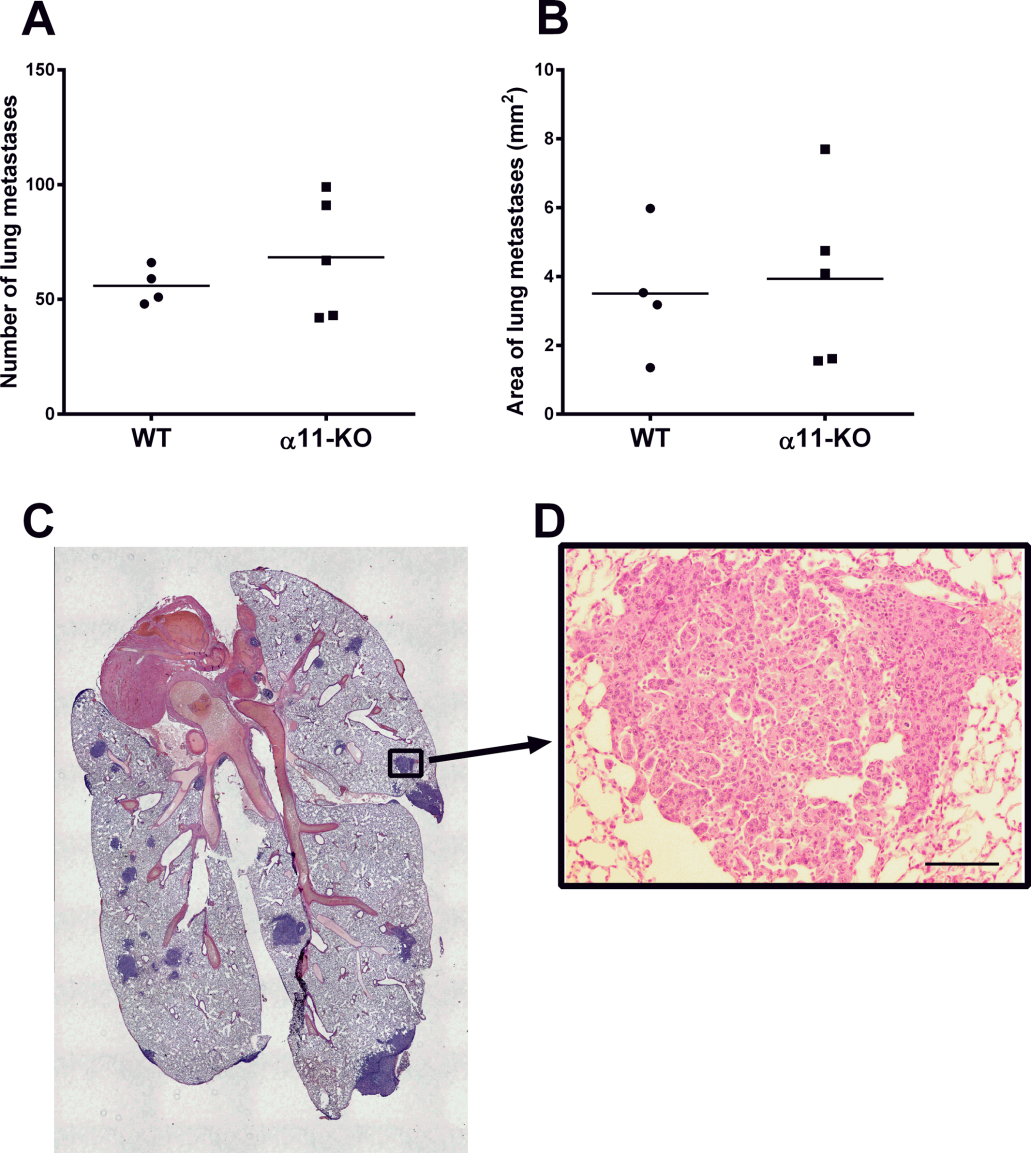
Histomorphometric quantification of H & E -stained lungs from the 4T1 model from SCID integrin α11 wild type (WT) (n = 4) and SCID integrin α11-deficient (α11-KO) (n = 5) mice. Average number of metastasis per section (A) and average area per section (B) is shown. The horizontal lines indicate the mean values. No significant differences were observed (p = 0.73 and p = 0.79) using Mann U Whitney t-test and unpaired two-tailed t-test, respectively. A representative lung with metastasis from a WT mouse is shown (C, D). Scale bar indicates 100 μm.

To evaluate whether stromal integrin α11 has an effect on metastatic potential, H & E stained sections from the 4T1 metastatic model were used. The 4T1 breasts cancer cell line is known to metastasize to lungs, liver, bone and brain [23]. Excessive macroscopic surface metastases were observed in all lungs. There was no difference in the 4T1 tumor cells ability to metastasize to the lungs in the α11-KO mice compared to the WT mice investigated at day 21 post injection (Fig 8). Metastases were also observed in the livers. In addition, the livers were significantly infiltrated by isles of extramedullary hematopoiesis, thereby making it difficult to quantify these metastases. Hence, the liver metastases were not further evaluated. No metastases were found in the brain of any of the animals at endpoint day 21.

## Discussion

Stromal integrin α11β1 has been implicated to play a role in experimental non-small-cell lung cancer (NSCLC) carcinogenesis [9, 10], and is expressed in human lung cancer [9, 17, 24] and in metastasis from human malignant melanoma [16]. However, the role of integrin α11β1 in other cancer types remains to be elucidated.

The present study showed that the primary tumor growth of RM11 prostate tumors was reduced in α11-deficient mice compared with WT mice, however, this was not the case in 4T1 mammary tumors. Furthermore, there was a shift towards thinner collagen fibrils in the 4T1 tumors grown in α11-deficient mice. In spite of altered collagen fibrils, there were no differences in the amount of activated fibroblasts, total collagen content, collagen organization or PIF in the tumors. In addition, the metastatic potential to the lung of 4T1 tumors was not affected by stromal α11-deficiency.

In this study we examined the role of integrin α11 in tumor stroma. Following injection of tumor cells in both WT and α11-KO mice, the implanted tumor cells derive their stroma from the host animal, and hence the tumors in α11-KO mice will have stroma that is deficient in integrin α11. Differences in tumor development can be presumed to be due to differences in tumor stroma between the two groups since the tumor cells injected are the same. As seen in Fig 1, we find that integrin α11 is expressed in tumors grown in WT mice, but not in the tumors grown in α11-KO mice.

There are only two previous *in vivo* studies concerning tumor growth and stromal integrin α11-deficiency, and they both showed a stimulatory effect of integrin α11 on tumor growth [9, 10]. First, wildtype mouse embryonic fibroblasts (MEFs) were found to have a greater stimulating effect on the growth of human NSCLC cells than MEFs lacking integrin α11 when co-injected in mice together with tumor cells [9]. Second, in a recent study by Navab *et al*., reduced tumor growth in α11-KO mice was described using subcutaneously implanted NSCLC xenografts [10]. In addition, in an *in vitro* heterospheroid model, Lu *et al*. showed that human lung cancer cells grown with WT fibroblasts had a higher tendency to proliferate and migrate compared to tumor cells grown with fibroblasts deficient in integrin α11 [18]. Taken together, these studies indicate that α11 integrin on fibroblasts interact with tumor cells and can play a role in regulating tumor growth. Nevertheless, in the present study we only found an effect on tumor growth in the subcutaneously implanted RM11 tumor model, and not in the orthotopically implanted 4T1 tumor model. It is not known whether these effects are tumor-type specific, or if other factors such as the location of tumor implantation, is of importance.

Integrin α11 is a collagen-binding integrin, and has previously been shown to play a role in collagen reorganization both *in vitro* and *in vivo* [10, 14, 15]. In this study we found that the collagen fibrils in the 4T1 tumors grown in integrin α11-deficient mice were thinner than those in tumors grown in WT mice. However, this finding was not universal, since the RM11 tumors showed no difference in collagen fibril diameter. A recent study on wound healing also found an increase in thinner collagen fibrils in the granulation tissue from integrin α11-deficient mice [25]. Furthermore, the study demonstrated that α11-deficiency resulted in reduced formation of granulation tissue and impaired wound contraction [25]. As tumors can be looked upon as “wounds that do not heal” and involve many of the same processes [26], this could represent an interesting parallel to the present study. It is somewhat surprising that in the current study the shift towards thinner collagen fibrils only was seen in the 4T1 model, in which there was no effect of integrin α11 deficiency on tumor growth, whereas no alteration in collagen fibrils was seen in the RM11 tumor model.

Activated fibroblasts play a crucial role in synthesis and remodeling of collagen in tumors [27, 28]. We found here that the amount of activated fibroblasts, identified by the marker α-SMA, was the same in tumors grown in α11-deficient mice when compared with WT mice. This does not correspond to the findings in the study on NSCLC where a decrease in α-SMA expression in α11-KO xenograft tumors was found compared to in WT xenograft tumors [10]. Moreover, reduced α-SMA expression was found in granulation tissue in integrin α11-deficient mice compared to WT mice [25]. In addition, also two earlier studies have suggested that integrin α11 stimulates fibroblast differentiation under different conditions [29, 30]. Thus, available data suggest that integrin α11 may play a role in activation of fibroblasts, although in this study we did not observe any difference in α-SMA expression in tumors grown in α11-deficient mice compared to WT mice.

Regarding the amount of collagen in the tumors, there was no significant difference in the amount of picrosirius-red staining in the 4T1 or the RM11 tumors in the present study. This does not correspond with the recent study on NSCLC where the amount of fibrillar collagen was reduced in xenografts in α11-KO compared to WT mice [10].

While we did not observe any difference in collagen organization in 4T1 or RM11 tumors, neither with SEM nor with picrosirus-red staining, Navab *et al* [10] found that collagen had a more non-linearized pattern in NSCLC tumors in α11-KO mice using different approaches, namely picrosirius-red staining, second harmonic generation imaging and atomic force microscopy. It is therefore likely that the role of integrin α11 in collagen organization differs in the NSCLC tumor model compared to the tumor models that were used in the present study.

Taken together, reduced tumor growth in α11-KO mice, has in an earlier study, been shown to be concomitant with reduced α-SMA and altered collagen structure in the tumors [10]. The findings in the present study, however, are different. Stromal α11-deficiency caused reduced RM11 tumor growth, despite no differences in collagen fibril diameter, collagen amount or α-SMA expression. In addition, there is a smaller proportion of activated fibroblasts (a-SMA staining) and collagen in the RM11 tumors than the 4T1 tumors (Figs 3 and 6), and one could therefore suggest that these stromal factors seem less relevant for RM11 tumor pathogenesis. Hence, in this study, the difference in tumor growth cannot be associated to changes in fibroblast activation or collagen alterations, and the pathogenesis behind the reduced tumor growth in the present study remains unknown. One can conclude, however, that the present findings indicate that different tumors seem to show different responses to stromal integrin α11-deficiency, although the mechanisms responsible are at this point not yet known.

One of the common features in the tumor microenvironment is the high interstitial fluid pressure (PIF), which can hinder efficient delivery of cytostatic drugs across the capillary barrier and into the tumor [31, 32]. Therefore, pharmacologically lowering of PIF can improve transport of cytostatic drugs [32]. Collagen in tumors has been recognized to be one of the factors important for tumor PIF [32–34]. Furthermore, two previous studies have shown that integrin α11 may have a role in regulating PIF in different conditions [15, 18]. It was of interest for us, therefore, to investigate the effect of stromal α11 deficiency on tumor PIF. In this study we did not find any difference in PIF between tumors grown in integrin α11-deficient mice compared to WT mice, indicating that α11 may not be important for PIF regulation in these tumors. Since collagen content is one of the factors that have been shown to be important for PIF [32–34], and the present study did not reveal any effect of stromal integrin α11 deficiency on amount of collagen in the tumors, an unchanged PIF was probably to be expected.

Little is known about how integrin α11 affects tumor metastasis, although there are some findings that indicate that it may play a role in the metastatic process. Integrin α11 mRNA has been found to be expressed in human metastases from malignant melanoma and high mRNA levels of the collagen binding integrins α1, α2 and α11 correlated with poor patient outcome [16].

In the *in vivo* study by Navab *et al*., NCI-H460SM lung carcinoma cells formed significantly less spontaneous metastases in SCID integrin α11-deficient mice compared to SCID WT mice [10]. Furthermore, in a heterospheroid *in vitro* model it was found that human lung cancer cells had reduced migratory and invasive capacity when cultured together with integrin α11-KO fibroblasts compared with WT fibroblasts [18]. Some studies have also indicated that integrin α11 on *cancer cells* may be important in the metastatic process, such as the study by Westcott *et al* where integrin α11 was among the genes highly expressed in a subpopulation of breast cancer cells with enhanced invasiveness [35]. Furthermore, two separate tumor studies indicate that integrin α11 RNA is upregulated during epithelial to mesenchymal transition (EMT) [36, 37].

However, in spite of earlier findings, in the present study we found no indication that there was a difference in lung metastases of 4T1 tumors grown in the integrin α11-deficient mice compared to those grown in WT mice, again demonstrating that integrin α11`s role in cancer development may vary in different tumor models.

## Conclusion

The present study showed reduced tumor growth in the α11-deficient mice in the RM11 model, but no effect in the 4T1 model, only partially confirming the suggested role of integrin α11 in promoting tumor growth. Even though the present study demonstrated an alteration in collagen fibril diameter in 4T1 mammary tumors in the α11-deficient mice, it did not confirm previously shown alterations in collagen amount and organization, or in α-SMA expression. These discrepancies may be due to differences in tumor model, tumor type, location and aggressiveness of the tumor type. Our findings clearly show that further investigations regarding the role of integrin α11β1 in different tumor types are needed.
